# Web-Based Health Coaching for Spinal Cord Injury: Results From a Mixed Methods Feasibility Evaluation

**DOI:** 10.2196/16351

**Published:** 2020-07-31

**Authors:** Sonya Allin, John Shepherd, Teri Thorson, Jennifer Tomasone, Sarah Munce, Gary Linassi, Christopher B McBride, Tizneem Jiancaro, Susan Jaglal

**Affiliations:** 1 Department of Physical Therapy University of Toronto Toronto, ON Canada; 2 Spinal Cord Injury BC Vancouver, BC Canada; 3 School of Kinesiology and Health Studies Queens University Kingston, ON Canada; 4 Toronto Rehabilitation Institute Toronto, ON Canada; 5 Department of Physical Medicine and Rehabilitation College of Medicine University of Saskatchewan Saskatoon, SK Canada

**Keywords:** community-based participatory research, spinal cord injury, self-management, motivational interviewing, internet-based intervention

## Abstract

**Background:**

Individuals with spinal cord injury (SCI) are at high risk of experiencing secondary conditions like pressure injuries. Self-management programs may reduce the risk of complications, but traditional programs have proven to be insufficiently tailored to the needs of people with SCI. To overcome barriers to self-management support, a web-based, self-management program was developed for Canadians with SCI called *SCI & U*.

**Objective:**

This study aims to evaluate the feasibility and potential impact of the *SCI & U* program in the context of a mixed methods pilot study.

**Methods:**

The study followed an explanatory, sequential mixed methods design. Participants (N=11) were Canadians with SCI who had been living in the community for more than 1 year. Each took part in a self-paced, six-session self-management program guided by a trained peer health coach. During sessions, participants could discuss a health topic with their coach from a predefined list (eg, skin or bowel management). Quantitative data were gathered before and after program participation to assess program feasibility and impact. Feasibility measures included attrition rates, frequency of topics selected, and recorded goals, whereas impact measures included measures of self-efficacy (University of Washington Self-Efficacy Scale [UW-SES]), mood (Personal Health Questionnaire Depression Scale [PHQ-8]), secondary conditions (Spinal Cord Injury Secondary Conditions Scale [SCI-SCS]), and resilience (Spinal Cord Injury Quality of Life Resilience Scale [SCI-QOL-R]). Qualitative measures were based on postintervention interviews; these were designed to confirm and expand on quantitative

**Results:**

Of the 11 participants, 10 completed pre- and postassessments, and 6 coaching sessions. Sessions lasted between 31 and 81 min (average 55, SD 13), and the duration of the program ranged from 35 to 88 days (average 56, SD 23). Diet and exercise were selected as topics 40% (20/50 sessions with topics) of the time, whereas topics such as mental health, bladder management, pain, and bowel management were chosen less frequently. Results gathered before and after the pilot study demonstrated improvements with moderate effect sizes on the UW-SES and the electronic health literacy scale (ie, Hedges g>0.5). Effect sizes for measures of resilience (SCI-QOL-R), depression (PHQ-8), and secondary conditions (SCI-SCS) were small (ie, Hedges g>0.3). Qualitative results confirmed a common focus on diet and exercise, and defined coaches as sources of accountability, information, reassurance and affirmation, and emotional and technical support.

**Conclusions:**

Results demonstrated that a web-based self-management program is feasible and acceptable by Canadians with SCI. Results also indicated a web-based, peer-led self-management program may impact resilience, self-efficacy, mood, and secondary complications. Finally, results illuminated the role of the coach in facilitating behavior change. Future work seeks to validate results in the context of a randomized controlled trial.

## Introduction

### Background

For individuals with spinal cord injury (SCI) who live in the community, the risk of experiencing secondary conditions such as pressure injuries or urinary tract infections is high. Even up to 20 years after the injury, rehospitalization rates for people with SCI remain over 30% [1]. To reduce the risk of secondary complications, people with SCI require knowledge about their injuries and strategies to mitigate risk. More specifically, knowledge and skills related to the management of “symptoms, treatment, physical, and psychosocial consequences and lifestyle changes inherent in living with a chronic condition” are required [2]. However, evidence suggests that knowledge and access to self-management tools are inadequate in the SCI community. For example, although some individuals with SCI report high levels of knowledge about bladder or pressure injury management on discharge from inpatient rehabilitation, studies indicate that less than half leave the hospital with clinically adequate knowledge [3]. Moreover, lack of knowledge is not the only barrier to self-care in the community; physical barriers, cost, lack of motivation, and pain also complicate the efforts to independently monitor and maintain health [4-6].

Self-management support programs are community-based tools designed to increase the knowledge and skills required to independently manage a chronic condition. As such, these programs carry the potential to fill some care gaps experienced by people with SCI in the community. Many existing programs, such as the Stanford Chronic Disease Self-Management Program [7] or the UK Expert Patient Programme [8], address a wide variety of chronic health conditions and are led by peers with the lived experience of a chronic condition. However, although peer-led programs have been associated with positive health outcomes (eg, improvements in self-efficacy, health-related quality of life [7,8], lower hospitalization rates [9], and reduced health care expenditures [10]), the typical formulation has shortcomings for community members with SCI. In one qualitative study of the CDSMP, for example, participants with SCI reported less program satisfaction than those with other conditions due to issues such as the lack of familiarity with SCI on the part of facilitators [11]. In this study and in another by Munce et al [12], individuals with SCI expressed a preference for web-based programming over in-person programming [11] and for content tailored to SCI management [11,12]. Tailored self-management programs delivered via the internet may therefore be met with higher satisfaction by individuals with SCI.

Community-based programs that deliver services to the SCI community via telephone or the internet have been both well received and are increasingly common in the literature [13-18]. Telephone-based self-management programs for people with SCI have proven to be safe and acceptable and to improve the participants’ level of activation and awareness [13-16]. Examples of internet-based health interventions include a nurse-led telehealth intervention that focused on newly discharged people with SCI [17], a tele-exercise intervention led by remote exercise coaches [18], and programs to support consultations with specialists [19] or peer educators [20] via iPads. However, internet-based self-management support for SCI, although associated with promising usability and feasibility results [19-21], are a relatively new concept and require further evaluation. The internet may indeed have special affordances for people with SCI; in randomized trials comparing internet and phone-based nursing interventions following inpatient rehabilitation, for example, internet-based programming is associated with the least number of postdischarge hospital visits [17]. Internet programming has also been identified as preferable to phone-based programming by members of the SCI community in a qualitative study by Munce et al [12].

In response to the interest in and need for web-based self-management support, a web-based program for users with SCI was developed at the University of Toronto. This program called *SCI & U* was modeled after telephone-based support programs (eg, SCI Action Canada [13,14] and My Care My Call [15,16]) and is part of a growing family of web-based support interventions for SCI (eg, PHOENIX [18-20] and SCI Health Storylines [21]). Like telephone-based interventions [13-16], the program consists of highly structured interactions between participants and trained peers with SCI, or *health coaches*. This is because, in the context of telephone-based programs, peer coaches have been perceived by participants as powerful motivators for behavior change and relevant, credible mentors for skill development [22]. Structures have been created to support participation by members of the Canadian SCI community in the program’s development [23]; these include a community advisory board and interdisciplinary design team. This kind of participatory approach reflects that of other research groups [13-16,20] and is intended to promote end user acceptance [24] and respect for guidelines for the creation of health programs serving individuals with disabilities [25].

### Objectives

This study aims to evaluate the *SCI & U* web-based self-management program in the context of a pilot study. The specific objectives were to assess implementation feasibility and the potential impact on self-efficacy and experience of secondary conditions. Evaluations of feasibility and impact were based on an analysis of quantitative data gathered before and after program participation. Qualitative data from postintervention interviews with the pilot participants were analyzed to validate and expand upon the quantitative findings.

## Methods

### Study Design

The study followed an explanatory, sequential mixed methods design with a quantitative component followed by a qualitative component [26]. The quantitative data were the first focus of the analysis, and the qualitative approach used was postpositivist in that the qualitative data did not serve to test the hypotheses but to create an understanding of the intervention that could be shared between the research team and participants [27]. The integration of quantitative and qualitative results took place at the interpretation and reporting levels. A joint display was created to relate the quantitative data to the relevant and explanatory quotes [28].

### Intervention

The *health coaching* intervention revolved around structured, web-based partnerships between participants and trained peer health coaches. In keeping with prior work [16], the intervention consisted of 2 principal components: trained peer health coaches and support materials.

A total of 5 peer health coaches were Canadians with SCI who acted as mentors and were trained in established health promotion techniques and paid as professional contractors. Peer mentoring after an SCI, on its own, has been associated with improved confidence in self-management skills and reduced hospital admissions [28]. However, trained peer health coaches have additional strengths as they have been educated in motivational interviewing (MI) [29] and brief action planning (BAP) [30]. Both MI and BAP dictate protocols that encourage health care recipients to actively participate in health care decisions [29,30]. Specifically, BAP helps health care recipients elicit, parameterize, and track realistic goals and plans for health behavior change [30]. It has been successfully embedded in many self-management programs [30], including *My Care My Call* [15,16].

**Figure 1:**
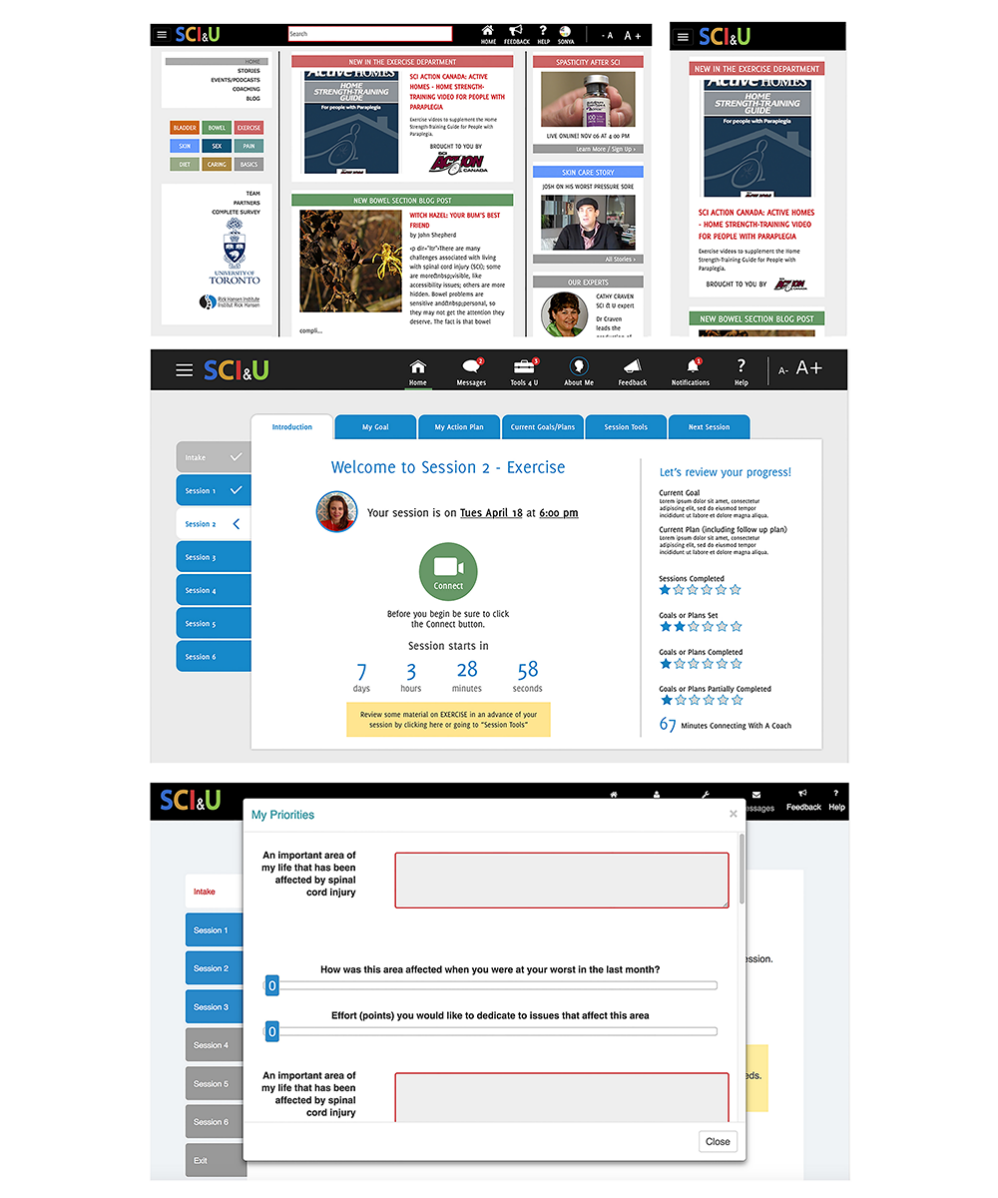
Top: the publicly accessible informational website that was designed to support the SCI & U program. In the middle, designs for the coaching dashboard of the secure platform. Bottom: a customized Patient Generated Index form, as implemented in the secure platform.

Web-based support materials were housed in a publicly accessible, curated information resource for SCI management [31], and a secure platform was designed to support confidential one-on-one interactions between the participants and web-based peer health coaches [32]. Both were developed using participatory processes [23]; the development of the coaching platform was additionally supported by an application program interface provided by a Toronto-based company called QoCHealth [33]. This allowed an in-house development team to create videoconferencing tools for Google Chrome browsers, tailored lists of topic-specific health information, and secure forms for goal setting and BAP (Figure 1). Of note, participants were not restricted in using alternative tools to communicate with coaches (eg, Skype) if Google Chrome was unavailable or these tools were familiar and comfortable to the participants. No conversations between the participants and coaches were recorded.

The consenting participants were recruited via community organizations and matched with the coaches based on their availability and the participants’ review of web-based coaching profiles and coach availability. The participants were matched with coaches with whom they had no relationship before the study. For the most part, the coaches worked with 2 participants at a time. Once partnerships were established, the participants engaged in up to 6 web-based self-management sessions with their coaches, using the coaching platform. The sessions were designed to last between 45 min and 1 hour, and pacing was flexible; however, the participants were asked to schedule sessions within a span of 2 weeks to complete all sessions within 12 weeks. The coaches were asked to dedicate as much as 1 hour of their time to prepare for each hour of interaction with their clients. The length of the sessions, flexible pacing, and program duration were informed by research detailing the relationship of these factors to the effectiveness of a telephone-based exercise intervention [14].

During the first *SCI & U* session, the participants worked with coaches to identify health priorities; these were recorded using a web-based version of the Patient Generated Index (PGI; Figure 1) [34]. The PGI allows individuals to formulate personalized goals and expectations for health care interventions; it has been used as an outcome measure in studies of chronic conditions and to articulate patient-centered treatment objectives [35,36]. At the end of each session, the participants were invited to select a single topic from a predefined list of health management *topics* for the subsequent session. The list of topics was informed by a prior survey of the Canadian SCI community [12]; these included management of pain, diet, exercise, mental health, bowel, bladder, skin, and sexuality.

During sessions, coaches discussed topics with participants and shared stories, listened, worked through issues, or helped formulate goals or plans according to the BAP process. Coaches were given scripts to guide conversations, but the precise content and structure of each session was directed by the participants. The scripts contained information standards for the MI and BAP processes, as well as topic-specific information drawn from a web-based educational website for the SCI community called SCI-U [37]. These educational materials were codeveloped and validated in the context of user studies by an author (JS) working alongside a team of Canadian SCI researchers and members of community organizations [38].

### Quantitative Component

#### Recruitment

The participants were restricted to English-speaking adults with traumatic, congenital, or nontraumatic SCI who had internet access and had been living independently in the community for at least a year. They were recruited by means of advertisements (tweets, flyers, and brochures) sent to members of organizations serving the Canadian SCI community. Additional recruitment efforts were led by a patient educator at the GF Strong Rehabilitation Centre in Vancouver, who introduced the study to the participants in outpatient programs. The study protocol was approved by both the University of Toronto (research ethics board [REB] #34808) and the University of British Columbia (REB #H17-01841). All participants provided oral consent and received an honorarium of Can $200 (US $150, Can $1 [US $1.33]) as compensation for the time required of them to complete the assessments.

#### Data Collection

Only participants, coaches, and the research team had access to the web-based coaching platform during the study. The quantitative data related to feasibility were derived from the participants’ attendance and usage of the coaching platform (ie, their completion of web-based activities). These measures included attrition rates for participants, the length of sessions, range of health management topics selected for discussion, and counts of goals and action plans established. In keeping with a related study directed at supporting self-management among inpatients with a mobile app, it was decided that the intervention would be considered feasible if more than 80% of the participants were retained for the duration of the study and if the same percentage adhered to the intervention [21]. For this prior study, however, adherence was related to usage of an app, whereas in this study, adherence was defined as having attended every web-based session with a coach. In addition, the number of goals and action plans established by participants and the variety of health topics they covered were considered indications of engagement, with more goals/plans and greater variety considered as markers of increased engagement.

The quantitative data to assess potential impact were derived from the participants’ independent completion of web-based assessments before and after participation. The assessment surveys were built into the platform and included measures of health-related self-efficacy, emotional and physical health status, and health literacy. In addition, the participants were asked to provide basic demographic information (eg, age, level of injury) via web-based surveys and before the commencement of coaching sessions. Initial surveys were completed within 1 week of each participant’s first session in the program; exit surveys were completed within 2 weeks of each participant’s last session.

Health-related self-efficacy is defined as the belief in one’s ability to meet, and overcome, health-related challenges to achieve the desired outcomes [39]. In this study, self-efficacy was measured using the short (6-item) form of the University of Washington Self-Efficacy Scale (UW-SES), a reliable measure validated in populations with SCI [40]. The responses were made on a 5-point Likert scale; higher scores indicate higher self-efficacy. In addition, resilience, or the ability to flexibly cope with challenges to independent health management, was measured using the short form of the Spinal Cord Injury Quality of Life Resilience Scale (SCI-QOL-R). This is an 8-item scale that has also been validated in SCI populations and that correlates with measures of depression, positive affect, and life satisfaction [41]. Similar to the UW-SES, responses on the SCI-QOL-R were made on a 5-point Likert scale; higher scores indicate greater resilience.

Physical health status was measured using self-reports on the Spinal Cord Injury Secondary Conditions Scale (SCI-SCS). This is a 16-item scale detailing secondary conditions associated with SCI, which impact health and functioning [42]; higher scores indicate more problems related to secondary conditions. The participants’ overall emotional status was measured using the Personal Health Questionnaire Depression Scale (PHQ-8), an 8-item scale commonly used to assess self-management interventions [43] and validated for use in the SCI population [44]. Scores on the PHQ-8 range from 0 to 24 and reflect recent experiences of depression; higher scores indicate more recent depressive symptoms [43].

Finally, to measure health literacy, the electronic health literacy scale (eHEALS) was used. This is a validated 8-item scale with questions related to skills at finding, evaluating, and using information found on the web to improve or maintain health [45]. In the SCI community, it has been used to evaluate the health information–seeking behavior of veterans with SCI [46].

#### Analysis

For all quantitative measures, we reported averages, SDs, and medians. The survey responses collected before participation in the program were compared with responses gathered after participation. To compare the pre- and postintervention scores in the surveys, we reported the results of two tailed, paired *t* tests. A significant change in scores was defined by *P* values under a Bonferroni-adjusted alpha level of .01 for each of the 5 outcome surveys (eg, .05/5) and power (ie, 1 minus the probability of failing to reject a false hypothesis) was set at 0.8. In addition, and because the sample sizes were small, we reported Hedges g statistics as estimates of effect size [47].

### Qualitative Component

#### Recruitment

In addition to the demographic and assessment surveys, the participants who completed the study were asked to complete semistructured one-on-one interviews following completion of the program. The purpose of these interviews was to validate the quantitative measures and expand upon them wherever possible, that is, shed light on the program mechanisms related to change in quantitative measures.

#### Data Collection

The consenting participants took part in the interviews, along with a member of the research team (either SM or JS). The interviews took place after the participants exited the program and via videoconferencing or phone. The interviews were recorded, transcribed, and deidentified; the analysis focused on the deidentified transcripts. To facilitate the organization and analysis of the deidentified data, NVivo software (QSR International) was used.

#### Analysis

The analysis followed an inductive thematic methodology, as described by Braun and Clarke [48]. The paradigm guiding the analysis was pragmatic and focused on the description of coaching experiences. It has been argued that approaches centering on descriptions of specific phenomena are suited to health services research, as they cater to both qualitative and quantitative analyses [49].

Overall, 4 authors (SA, TT, JS, and SM) met several times to discuss and assign codes to the transcript data. The group then proposed a coding framework. This was iteratively refined and, once finalized, was applied to a subset of interview transcripts by 2 team members (SA and TT). The coders worked together to guarantee reliable intercoder agreement on the subset and suggest new themes or refine existing themes. A total of 4 members of the research team (SA, TT, JS, and SM) reviewed all the proposed refinements. After a consensus was reached, the final coding framework was applied to all transcripts by the lead author (SA).

After coding, the relationships between the codes were discussed and analyzed using Jaccard similarity coefficients [50]. The Jaccard coefficients required each code to be presented as a vector of ones and zeros, where each element in the vector reflected the presence or absence of a specific transcript. It was suggested that pairs of codes with high coefficients were relatively likely to co-occur in transcripts. However, all quantitative suggestions of coding associations were manually reviewed to guarantee validity.

## Results

### Participant Demographics

Table 1 provides details of the demographics of participants. A total of 11 participants took part in the pilot, including 7 women and 4 men. Of the 11, 7 were from relatively rural Ontario (ie, municipalities with populations under 100,000); others were from the Vancouver area in British Columbia. Traumatic SCI was reported by 8 participants. Of these, 3 reported nontraumatic SCI, and 1 reported spina bifida. The participants who took part in the pilot reflected a mix of gender, level of injury, injury type, and use of mobility aids. All had been living with SCI for >5 years, with an average of 20 years and a median of 22 years. Most reported that they were regular users of both the internet and had access to email; several additionally reported regular use of social apps (eg, Facebook) and YouTube or games.

Of the 11 consenting participants, 10 completed web-based baseline assessments, and 9 completed both baseline and follow-up assessments. One participant was not comfortable using the web-based coaching platform, so the research team deferred to telephone delivery of the coaching service for this person. Although she completed her initial visit via videoconferencing software, this required the involvement of a personal support worker, and the telephone was therefore seen as preferable. This participant did not complete any web-based activities or assessments, so her data were excluded from the quantitative analysis of impact and feasibility.

**Table 1:** Demographics of participants (N=11).

Characteristics		Values
Age^a^ (years), mean (SD)		43 (8)
Years since injury^b^, mean (SD)		20 (12)
**Gender, n (%)**		
	Male	4 (36)
	Female	7 (64)
**Province of residence, n (%)**		
	Ontario	7 (64)
	British Columbia	4 (36)
**Mobility, n (%)**		
	Powered wheelchair	6 (55)
	Manual wheelchair	3 (27)
	Walker	1 (9)
	Not reported	1 (9)
**Injury etiology, n (%)**		
	Traumatic	8 (73)
	Nontraumatic	3 (27)
**Injury level, n (%)**		
	Paraplegia	5 (46)
	Tetraplegia	3 (27)
	Not reported	3 (27)

^a^Median age was 46 years.

^b^Median number of years since injury was 22 years.

### Quantitative Results

#### Feasibility

One participant completed baseline assessments only to later withdraw from the study due to the birth of her child. All remaining participants (n=10) completed 6 sessions with their coach; 9 used the web-based platform, and 1 used the telephone. However, the participant who used the telephone did not complete the web-based intake or exit surveys. The resulting retention rate for the pilot was 10 of 11 (81%), and the adherence rate was 100%, but substantial data were missing for 1 of 10 (10%) remaining participants. The participants who accessed the web-based surveys were able to successfully complete these most of the time; complete data for ≤7 out of the 9 participants were recorded for every study measure. All sessions were conducted between December 2017 and April 2018.

Across the 10 participants who completed the program, the median session duration with a coach was 55 min (range 31-81 min). Most participants completed the sessions within 14 days of each other (the average interval between the sessions was 11 days; range 3-45 days). There was variation in the overall duration of the program for participants: the average program duration was 56 days with a range of 35 to 88 days. The coaches reported frequent rescheduling of sessions due to participant illness or issues coordinating across time zones, but the exact number of rescheduled sessions was not captured during the pilot. The coaches were asked to initiate the rescheduling of the missed sessions, wherever possible.

Although the participants were invited to use the coaching platform’s built-in videoconferencing software, most defaulted to communication tools with which they were familiar with. A total of 2 participants used the built-in videoconferencing tool initially, later defaulting to the use of the Zoom videoconferencing platform [51]. There were 5 others using the Zoom platform exclusively, 1 participant used Skype [52], and 1 used Facebook Messenger [53]. This occurred because the built-in tool was relatively limited; it was optimized for Google Chrome specifically and would not work on some devices (eg, iPads). The one participant who did not use the web-based platform initially met her coach via Skype because technical support (a personal support worker) was available at her home at that time. Thereafter, she defaulted to communicating with her coach by telephone.

The participants elected to discuss a health management topic with their coach most of the time; only 7 sessions (or 14% of the 50 sessions following the first) were left open ended or without a *topic* from the list of predefined choices. Many participants selected a different topic for every session; however, 1 participant elected to focus on *diet* and another on *exercise* for 3 sessions in sequence. Goals or action plans were established during 30% of the sessions, but there was significant variation in the participants’ goal-setting behavior. Some elected to establish new goals or action plans at every session, whereas others never set a goal. Established goals and plans were also frequently unrelated to the health management *topic*, chosen for a given session. The topics selected by the participants and the examples of goals/action plans are provided in Table 2.

**Table 2:** Health management topics chosen by participants for coaching sessions and related goals.

Session topics	Total sessions, n (%)	Total participants, n (%)	Example goals [participant ID]
Diet	11 (22)	9 (90)	“To plan, prepare and cook more nutritious meals myself.” [Number 6] “Healthy Food/fruits and vegetables/stay away from carbs.” [Number 5]
Exercise	9 (18)	7 (70)	“Go in to [a community recreation facility] and get set up for family plan.” [Number 3] “You will do your exercises [arm bike] and stretches that your occupational therapist has suggested.” [Number 4]
Mental health	8 (16)	8 (80)	“To find someone to talk to about frustration & depression.” [Number 1] “To talk with my husband and son about my frustration and need for them to contribute more.” [Number 2]
Other	7 (14)	5 (50)	“To buy a handicap accessible van.” [Number 2] “Study material for direct funding 45 minutes daily, 7 days/week.” [Number 7] “Take a rest/nap for at least an hour every other day.” [Number 8] “Getting the wheelchair done/be in touch OT and seating specialist.” [Number 5]
Bladder	6 (12)	6 (60)	“Maintain the status quo with my bladder until I see the surgeon and the urologist.” [Number 2] “Drink more than 8 glasses a day [of water].” [Number 5]
Pain	5 (10)	5 (50)	No goals related to pain management were recorded
Bowel	4 (8)	3 (30)	“More fiber intake.” [Number 5]
Skin	1 (2)	1 (10)	No goals related to skin management were recorded

### Impact

Baseline assessment data are shown in Table 3. It may be noted that 2 participants reported PHQ-8 scores consistent with major depression at the outset of the pilot (ie, their scores were >10), and on the SCI-SCS, more than two-thirds reported that they had moderate or significant recent problems with urinary tract infections, circulation, spasticity, or pain. Recent moderate or significant bowel, bladder, and sexual dysfunction were also reported by more than half of the participants.

The quantitative results related to changes in measures before and after the pilot are also given in Table 3. The graphs detailing baseline scores by changes in scores for each outcome measure have been provided in Multimedia Appendix 1. Positive postintervention changes were associated with all outcome measures, and effect sizes for the UW-SES and eHEALS were moderate (ie, >0.5). Effect sizes for the measures of resilience (SCI-QOL-R), depression (PHQ-8), and secondary conditions (SCI-SCS) were small (ie, >0.3). No changes were found to be significant, although changes in SCI-QOL-R scores neared significance (*P*=.08).

A closer inspection of the scores on the SCI-SCS subscales indicated larger effects related to bowel dysfunction and pain subscales of the SCI-SCS. The estimate of effect size for self-reports on the pain subscale based on Hedges g was moderate at 0.52 (95% CI 0.01 to 1.04), whereas that of the bowel dysfunction subscale was large at 1.04 (95% CI 0.04 to 2.11).

**Table 3:** Pretest and posttest measures.

Scale	Maximum value	Values, n (%)	Pretest		Posttest		Paired *t* test *P* values (adjusted)	Hedges g (95% CI)
			Mean (SD)	Median	Mean (SD)	Median		
UW-SES^a^	30	9 (90%)	19.2 (6.3)	21.3	23.2 (2.9)	24.0	.33	–0.52 (–1.08 to 0.03)
SCI-QOL-R^b^	40	9 (90%)	29.3 (5.7)	27.0	31.7 (4.6)	31.0	.08	–0.36 (–0.61 to –0.10)
SCI-SCS^c^	48	7 (70%)	21.7 (7.2)	22.0	17.9 (6.4)	18.0	.71	0.48 (0.02 to 0.94)
PHQ-8^d^	24	8 (80%)	7.75 (5.2)	5.0	6.0 (3.5)	4.5	.27	0.30 (–0.11 to 0.71)
eHEALS^e^	50	8 (80%)	37.1 (4.7)	37.5	40.8 (6.2)	41.5	.43	–0.59 (–1.29 to 0.10)

^a^UW-SES: University of Washington Self-Efficacy Scale.

^b^SCI-QOL-R: Spinal Cord Injury Quality of Life Resilience Scale.

^c^SCI-SCS: Spinal Cord Injury Secondary Conditions Scale.

^d^PHQ-8: Personal Health Questionnaire Depression Scale.

^e^eHEALS: electronic health literacy scales.

### Qualitative Results

**Figure 2:**
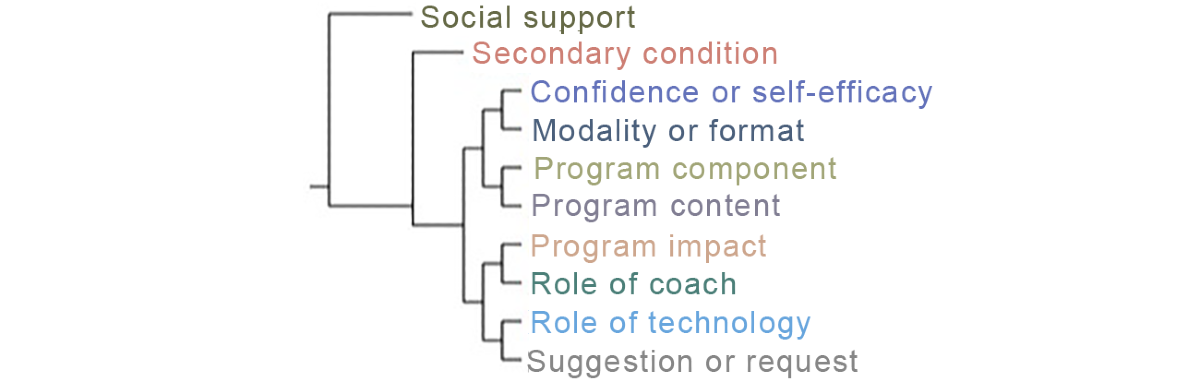
Tree Map of codes as generated by NVivo software. Codes that are close together are those with high Jaccard coefficients.

The coding dictionary that was agreed upon by the 4 authors (SA, JS, TT, and SM) is presented in Multimedia Appendix 2. A tree map illustrating codes as they were applied to transcripts is shown in Figure 2.

The following results focused on the theme of “Program Impact,” as the statements related to this theme were able to directly validate and expand upon the quantitative data. In addition, we focused on the “Role of Coach” as this theme was frequently colocated in the transcripts alongside the participants’ statements about program impact.

#### Program Impact

Similar to the quantitative data related to the frequency of the selected health topics, the qualitative data confirmed diet and exercise as having been discussed with the coaches by many participants. Descriptions of program-related dietary changes were common; the examples documented in the interviews included experimentation with gluten-free and fiber-rich diets. Changes in fluid intake were also recorded; this was specifically related to bladder health during the interviews. It may be noted that although some diets influenced the experience of bowel dysfunction related to SCI [53], bowel management was relatively infrequently selected by participants as a guiding topic for the discussions.

Along with the changes in diet, changes related to exercise were also frequently described by the participants. This confirms that quantitative data indicating exercise was a popular topic. Examples of exercise-related changes drawn from the interviews included speaking to physicians about exercise routines, joining a gymnasium, and contacting a personal trainer. The participants described the impact of these changes as having a wide range. Participant number 3, for example, related the fact that she was “moving more” to her experience of pain and decrease in opioid use. This participant also noted that her family relationships had improved because they “all benefitted” from a membership to a local community center. Others indicated changes in exercise routines improved mood. Changes to exercise routines may therefore be related to changes in other quantitative measures, such as, the SCI-SCS and PHQ-8.

Some participants reflected on their discussions with the coaches about mental health. This reflects that quantitative data indicating mental health was selected as a topic about 16% of the time. Overall, 2 participants specifically referenced the impact of weather on their mental health and described their interactions with the coaches as particularly meaningful during the winter months. It may be noted that the program was conducted during the winter for all participants.

A joint display relating the participants’ impact statements to the quantitative findings is presented in Table 4.

**Table 4:** Joint display of impact statements made by participants alongside quantitative results.

Topics	Quantitative measures	Selected quotes from interviews [participant ID]	Meta-inference
Bowel and bladder management	SCI-SCS^a^ bowel subscale (Hedges g=1.04): 18% of the sessions on diet, 10% on bladder, and 8% on bowel	“Part of the conversation got me to go to my doctor and ask about gluten free diets.” [Number 5]) “[The program] certainly improved my eating habits.” [Number 3]) “I’m always having a hard time digesting … [my coach and I] would talk about that.” [Number 5]) “When we were doing … kidney and bladder, I really sort of upped my fluid intake.” [Number 1]	Several participants described dietary changes resulting from the program; the impact on bowel dysfunction may be indirectly related to these changes
Exercise	SCI-SCS (Hedges g=0.48): 11% of the sessions on exercise	“I had been meaning to contact a trainer at a gym here …. I texted during [my coaching] session and she messaged me back right away.” [Number 2] “I have a real hard time sleeping. We thought, you know, maybe exercise [could help].” [Number 1]) “[The program] pushed me to do exercises more.” [Number 7]	Many participants described changes to exercise routines resulting from the program; the changes were designed to influence diverse health issues, such as sleep. The overall changes on the SCS may be related to both changes in diet and exercise
Pain	SCI-SCS pain subscale (Hedges g=0.52): 10% of sessions on pain	“I’m moving more. I’ve reduced my opioids right down to nothing and I’m at a two-week point there.” [Number 6]	One participant specifically reported exercise as having impacted pain and the use of pain medication
Mental health	PHQ-8^b^ (Hedges g=0.30): 16% of sessions focused on mental health	“[We discussed] depression. You know, in the winter, obviously, we all get a little bleak, not being able to get outside, so that was a good [topic] at that time.” [Number 8] “I’ve stayed stable … I’m in a better place. I feel good mentally.” [Number 6]	Mental health was a frequent topic of discussion; some participants specifically related changes in mood to changes in exercise

^a^SCI-SCS: Spinal Cord Injury Secondary Conditions Scale.

^b^PHQ-8: Personal Health Questionnaire Depression Scale.

#### Role of the Health Coach

Alongside the discussions regarding program impact, the participants made frequently mentioned the supportive roles played by their coaches. The coaches were credited as being sources of accountability, inspiration and encouragement, social support, and health management information. In addition, the coaches were frequently credited as being sources of technical support. These roles are described in greater detail in the following sessions.

##### Source of Accountability

The participants frequently mentioned the fact that the program had increased their accountability to plans for behavior change, and that their coach had played a critical role in fostering this accountability. Being available to listen and check in on plans motivated many participants to take action. The coaches were also described as affirming and bolstering the participants, even when plans did not go as expected:

He [said] “what are your goals this week” and “I’ll check up on you in the next week or in two weeks and we’ll talk about it”. So, every conversation was like, “hey, I did this and I accomplished this”, which made it that much more interesting to get back to each other and talk.

Number 2

##### Source of Inspiration and Encouragement

Coaches were often described as acting like “cheerleaders” [Number 1] and as valuing the participants’ efforts, regardless of the outcome. In addition, many participants described the coaches as role models. Some specifically related their self-management behaviors to those of their coaches:

My coach… has low-level quadriplegia and here I am with paraplegia. I felt if anything, my gosh, why am I being so whiny about being active when … [my coach] would have a more difficult time being active?

Number 3

##### Source of Information

The coaches were often credited as sources of health information. Several times, the participants described working with the coaches to search for web-based information or receive useful links in their email from the coaches after a session. Sometimes, information flow was described as going both from coach to participant and vice versa:

We had some good conversations each week for each [self-management] topic. [My coach] made some recommendations on the last session that I haven’t had a chance to look at and do yet, but I will.”

Number 7

[My coach] was always really good at sending me things to read. Whatever we talked about there was always a follow-up to read.

Number 6

##### Source of Social Support

Interactions with coaches were often valued as social support. Many described the support of peer coaches as particularly meaningful during the winter and due to a perceived level of mutual understanding:

I get feeling stuck a lot or like I’m alone. I have a service dog, but he only does so much. So, yeah, [it helps] just to know that I’m not alone.

Number 1

[My coach] was good, because …. there are some similarities in his life … so he could relate on the same level as me.

Number 5

##### Source of Technical Support

Finally, although the coaches were primarily focused on supporting the participants’ self-management efforts, they were frequently asked to troubleshoot technical issues for participants. The commonly encountered technical issues related to videoconferencing software (either built into the platform or from a third party) and web-based forms for action planning or assessment:

When I did the intake, [my data] didn’t save … so I had to go through with my coach.

Number 3

My coach… had to spend a bit of time guiding me as to where [to enter my goal] … I think once she may have even entered in the goals for me because I couldn’t figure out where I was supposed to.

Number 1

## Discussion

### Principal Findings

The results indicated that the delivery of a web-based self-management program tailored for the Canadian SCI community is feasible, and that its use may be associated with positive changes in terms of self-efficacy, mood, resilience, and experience of secondary conditions. The qualitative and quantitative results illustrated the wide variety of self-management topics touched upon by the intervention and the range of self-reported behavior changes adopted as a result. In addition, the qualitative results shed light on the specific mechanisms used by the coaches to promote behavior change; these included provision of encouragement, information, social support, and accountability.

### Comparison With Prior Work

The feasibility results, in terms of adherence and retention, reflected the preliminary work related to the development of other web-based self-management programs for the SCI community, such as PHOENIX [20] and SCI Health Storylines [21]. A feasibility study evaluating the use of SCI Health Storylines by 20 individuals with SCI found that 85% were retained as inpatients and 70% were retained at 3 months post-discharge [21]. Our results were similar, as 81% of the participants remained throughout the pilot. However, the adherence measures for the two studies were not directly comparable, as SCI storylines did not require attendance at the scheduled web-based sessions with a coach. Instead, our adherence measures better reflected those from a tele-exercise intervention for the SCI community, which, like ours, reported 100% adherence [18]. This same study reported that 85% of the exercise data were successfully recorded by the developed technology; in this pilot, the data were successfully collected from 9 of 10 participants, and each study measure was completed by at least 7 of the 9 participants.

In terms of technology usability and acceptance, the results reflected those of the PHOENIX study, which demonstrated iPads and iTunes U to be acceptable for peer-led education and self-management support among users with SCI [20]. A related study exploring the use of iPads to facilitate periodic web-based medical consultations between users with SCI and health professionals revealed similarly high user acceptance of tablets and an overall preference by participants for the web-based, rather than in-person or telephone-based meetings [19]. Our results echoed the same in the context of an intervention spanning multiple sessions. In addition, most participants in this study were able to satisfactorily complete web-based activities; only one deferred to use of the telephone to complete activities such as goal setting. Both this study and the PHOENIX study illustrate participants’ relative comfort with mainstream, community-based videoconferencing tools (eg, Skype, Face Time, and Zoom) [20]. Although this study provided custom videoconferencing tools, most deferred to tools with which they were familiar, even despite potential failure to comply with Canadian Health Information Privacy Policies (eg, Personal Health Information Protection Act).

The topic of the use of videoconferencing software in the context of community-based research projects like this one is an increasingly controversial topic, particularly in the light of revelations regarding potential security loopholes [54]. Given that videoconferencing data were not collected by the research team and security risks were not well understood, research partners in this study permitted deferral to communication tools to promote accessibility. The built-in tool was, as mentioned previously, limited in that it was optimized for Google Chrome and would not work on other devices such as iPads. The issue has since been discussed with the University of Toronto REB, which is now indicating that it will explicitly recommend the use of institutionally sanctioned software (eg, Skype for Business [52]) for research communication and will ask research teams for justification to use alternatives (eg, Zoom [51]) in future studies. Moreover, these policies are actively changing as new risks are exposed. Moving forward, the SCI & U team has chosen to focus on informing the participants about potential security risks related to web-based communication, and our protocol is being amended to reflect this change in practice. The practices will likely continue to change as the situation evolves and policy comes to light.

The feasibility results also demonstrated user engagement and highlighted the variety of ways in which participants chose to tailor the SCI & U program. Although diet and exercise were the most popular topics of discussion during the coaching sessions, the participants also chose to discuss issues such as pain, bowel dysfunction, and sex. Flexibility was found to be similarly valuable in the context of the *Get in Motion* intervention. Participants in this study were found to have very different needs in terms of program pacing and duration based on their baseline levels of activation, for example [14]. In addition, the users in this study elected to set goals 30% of the time, indicating engagement with program activities.

In addition, both this intervention and others [15,16,20] illustrate the feasibility of participatory approaches to program development. For the PHOENIX program [20], a community-based *task force*, including members of the SCI community, worked to identify the needs, develop digital content for, and provide feedback on the program as it was developed. In this study, a similar task force was asked to iteratively comment on program content and design [23].

Perhaps, the impact results might best be related to those from a recent randomized controlled trial (RCT) of the *My Care My Call* program [15]. This was a peer-led, self-management support intervention targeting the management of secondary conditions delivered to 84 participants with SCI over 6 months. As with SCI & U, peer health coaches were trained in the use of both the MI and BAP techniques. The results showed that program participation was associated with a significant increase in measures of activation, resource awareness, social participation, and quality of life. In this study, program participation was associated with similar improvements in the measures of self-efficacy and resilience and in the ability to navigate electronic health resources.

Fewer studies have measured the health outcomes resulting from self-management interventions in the SCI population, and none that we know of are built around web-based interactions with trained peers and associated activities. However, for newly discharged people with SCI, peer mentoring has been associated with a reduced number of return hospital visits after 6 months [55]. The participation by individuals with SCI in peer-led exercise programs has been associated with improved self-reported physical activity 6 months after program participation [13]. Similar exercise outcomes, in turn, have been associated with decreased pain and improved mental health [56,57]. This study focused on relatively short-term outcomes (<12 weeks) and measured the experience of secondary health complications based exclusively on self-reports on the SCI-SCS. Nevertheless, the results pointed to potential moderate effect sizes on this scale, with larger effects on specific subscales of the SCI-SCS (eg, bowel dysfunction and pain). Some effects might have been incidental to the changes in diet or exercise as such changes were widely reported by the participants during the interviews.

The qualitative results illustrated the variety of ways in which coaches supported the participants’ health-related behavior change. The specific roles taken up by the coaches echoed those taken up by the coaches of the *My Care My Call* program. A content analysis of more than 500 *My Care My Call* telephone calls found the coaches to have fostered behavior change by acting as *role models*, *advisors*, and *supporters* [22]. The same study found the role of *advisor* as reflecting the coaches’ training and expertise in the MI and BAP techniques. The coaches in this study also acted as *role models* or *supporters* and served as *advisors* when they walked the participants through the BAP protocol or provided information. However, the coaches of SCI & U were additionally asked to act as *advisors* when they provided technical support. Technical requests might indicate a somewhat expanded role of *advisor* in the context of programs like SCI & U.

### Limitations

The results were based on a limited sample. This was a small feasibility study with a convenience sample of 11 participants from two Canadian provinces. The individuals who responded to the recruitment calls might have been people with both motivation and internet access a priori and therefore biased. Moreover, the majority of the participants experienced injuries more than 10 years before commencement of the study.

The quantitative outcome measures were based on self-reports and were only collected at baseline and as participants exited the program. It remains unclear whether the changes reported by the participants were or will be robust to time.

In addition, this study lacked a control group. Confounding factors, such as the weather, were not controlled and might have influenced outcome measures. The impact of the weather on the participants’ mood was, in fact, referenced during the interviews.

Moreover, the results presented in this study omitted the perspective of the coaches in the study on issues such as program feasibility (eg, its demands on their time and their experience of the technology). However, the perspectives of coaches were collected through postintervention interviews that are currently being analyzed. We expect a future study to touch upon the impact and feasibility of the program as perceived by these individuals. To validate the results and ensure that they are robust to both time and confounding factors, the intervention is currently being tested in the context of a longer RCT.

### Conclusions

A web-based self-management program that has individuals with SCI partnering with trained peers has been demonstrated to be feasible. Moreover, the program is a promising means to develop self-efficacy, health literacy, and resilience in a population at high risk of health complications while living in the community. These results indicate the potential of web-based tools to bolster existing community-based support for people with SCI, particularly for those in small or rural communities.

Future work seeks to validate the results of this pilot study. An RCT is currently underway for this purpose; this will compare the effects of the SCI & U intervention over the span of a year on an experimental group and relative to wait-listed controls.

## Appendix

Multimedia Appendix 1 Baseline scores by change in scores (ie, follow up score minus baseline) for measures on the PHQ-8, SCI-SCS, eHEALS, UW-SES and SCI-QOL-R.

Multimedia Appendix 2 The coding dictionary agreed upon by authors for coding of transcripts.

## Abbreviations

BAP: Brief Action Planning
eHEALS: electronic health literacy scale
MI: motivational interviewing
PGI: Patient Generated Index
PHQ-8: Personal Health Questionnaire Depression Scale
RCT: randomized controlled trial
REB: research ethics board
SCI: spinal cord injury
SCI-QOL-R: Spinal Cord Injury Quality of Life Resilience Scale
SCI-SCS: Spinal Cord Injury Secondary Conditions Scale
UW-SES: University of Washington Self-Efficacy Scale
