# Supplementary figures and images for: Web-Based Health Coaching for Spinal Cord Injury: Results From a Mixed Methods Feasibility Evaluation
Source: JMIR Rehabil Assist Technol. 2020 Jul 31;7(2):e16351. doi: 10.2196/16351 (PMC7428932; doi:10.2196/16351)

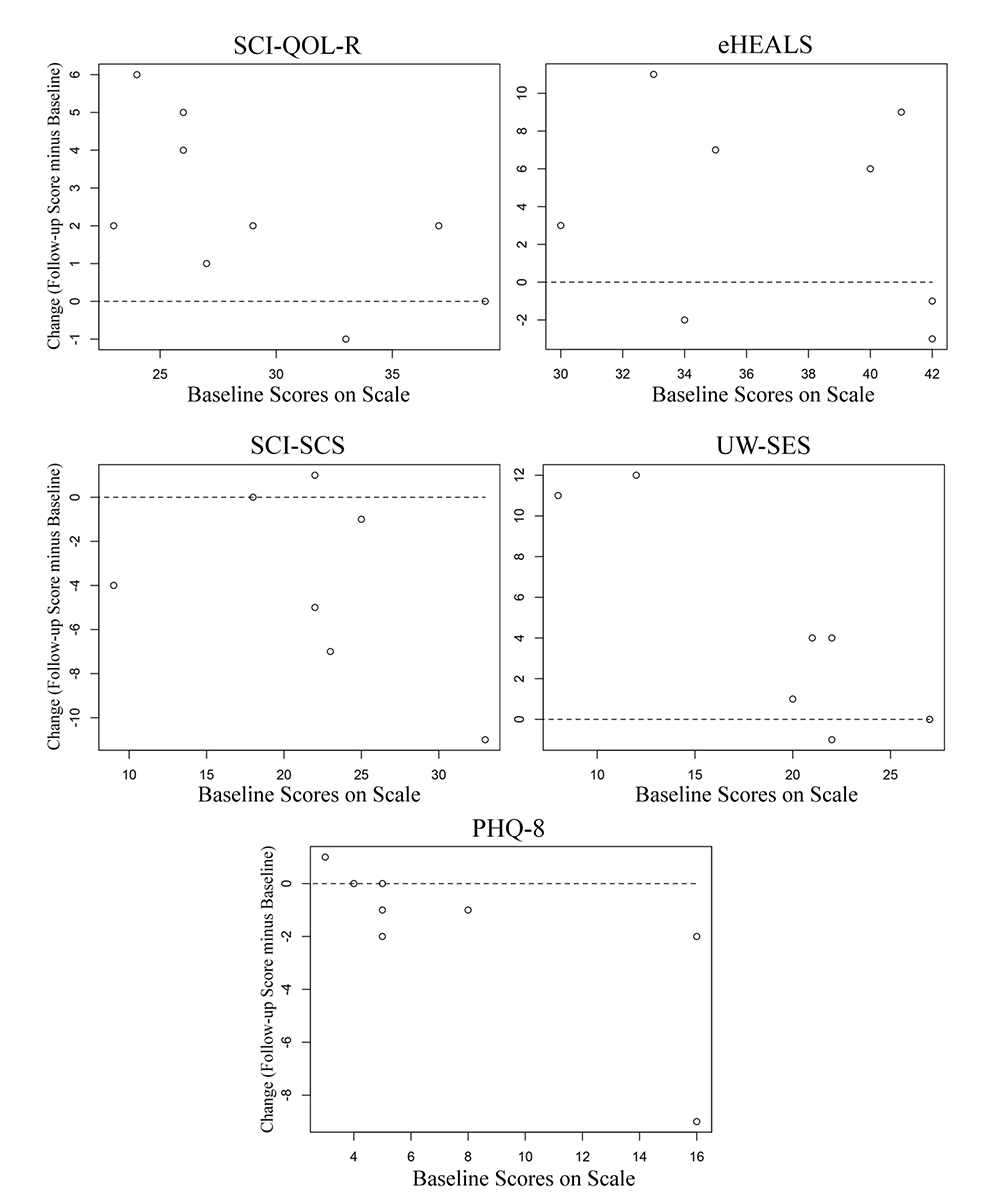

Supplement: Multimedia Appendix 1 [file rehab_v7i2e16351_app1.png]
