# Supplementary material for: Web-Based Health Coaching for Spinal Cord Injury: Results From a Mixed Methods Feasibility Evaluation
Source: JMIR Rehabil Assist Technol. 2020 Jul 31;7(2):e16351. doi: 10.2196/16351 (PMC7428932; doi:10.2196/16351)
Supplement: Multimedia Appendix 2 [file rehab_v7i2e16351_app2.docx]

**Appendix 2: Coding Framework**

1. Modality/Format: Meaning the preferred format, technology or protocol used during coaching sessions. Could refer, for example, to a preference for group over 1 on 1 meetings or for a preference for Google Chat sessions over Skype.
2. Program Content: Meaning the information discussed by participants with coaches as a part of the program, with a focus on “topics” discussed or reviewed during sessions (e.g. Bowel Management).
3. Program Component: Discussion that explicitly refers to application of MI or BAP techniques (e.g. action planning, goal setting, shared stories) - i.e. specific skills trained in coaches and delivered as part of program.
4. Program Impact: Meaning the perceived impact of the program on the participant, which could be related to mood, to an exercise routine or effect on a secondary condition, for example.
5. Role of Coach: Meaning the perceived services and functions of the coach (i.e. as a confidante, as a mechanism to promote accountability). Can include discussion of coaching relationships, the perceived expertise of the coach, or of the character of the match.
6. Secondary Condition: Code to use for any specific discussion of a secondary condition experienced by the participant (e.g. pain or bladder management), or treatment related to a secondary condition (e.g. going to see the urologist).
7. Confidence/Self-Efficacy: Code to be used on any passage that illustrates feelings of empowerment on the part of discussants (or lack thereof) and as related to management of health.
8. Suggestion/Request: Code to be used for discussions of problems encountered and to record suggestions for potential program changes (i.e. addition or deletion of a feature or element).
9. Role of Technology: Code for descriptions of technology, either positive or negative.  E.g., I enjoyed the links, the page did not save, it was convenient to use the webcam, etc.  Anything related ease of use (or lack thereof).
